# Glioblastoma radiotherapy using Intensity modulated Radiotherapy (IMRT) or proton Radiotherapy—GRIPS Trial (**G**lioblastoma **R**adiotherapy via **IMRT** or **P**roton Beam**S**): a study protocol for a multicenter, prospective, open-label, randomized, two-arm, phase III study

**DOI:** 10.1186/s13014-021-01962-8

**Published:** 2021-12-20

**Authors:** Laila König, Cornelia Jäkel, Nikolaus von Knebel Doeberitz, Meinhard Kieser, Fabian Eberle, Marc Münter, Jürgen Debus, Klaus Herfarth

**Affiliations:** 1 Department of Radiation Oncology, University Hospital Heidelberg, Im Neuenheimer Feld 400, 69120 Heidelberg, Germany; 2 Heidelberg Institute of Radiation Oncology (HIRO), Heidelberg, Germany; 3 Division of Radiology, German Cancer Research Center (DKFZ), Heidelberg, Germany; 4 Institute of Medical Biometry, University of Heidelberg, Im Neuenheimer Feld 130.3, 69120 Heidelberg, Germany; 5 Department of Radiation Oncology, University Hospital Marburg/Gießen, 35043 BaldingerstraßeMarburg, Germany; 6 Marburg Ion-Beam Therapy Center (MIT), Department of Radiation Oncology, Marburg University Hospital, Marburg, Germany; 7 Department of Radiation Oncology, Hospital Stuttgart, Stuttgart, Germany

**Keywords:** Proton radiotherapy, Glioblastoma, RANO, Toxicity, Chemoradiation

## Abstract

**Background:**

Radiation therapy is an integral part of the multimodal primary therapy of glioblastomas. As the overall prognosis in this tumor entity remains unfavorable, current research is focused on additional drug therapies, which are often accompanied by increases in toxicity. By using proton beams instead of photon beams, it is possible to protect large parts of the brain which are not affected by the tumor more effectively. An initial retrospective matched-pair analysis showed that this theoretical physical benefit is also clinically associated with a reduction in toxicity during therapy and in the first few months thereafter.

**Methods/design:**

The GRIPS trial is a multicenter, prospective, open-label, randomized, two-arm, phase III study using either intensity modulated photon radiation techniques (standard arm) or proton beam radiotherapy (experimental arm). Additionally, patients are stratified according to "fractionation scheme" (normofractionated/hypofractionated), "subventricular zone involvement" (yes/no) and concurrent chemotherapy (yes/no) and the planned case number is 326 patients.

Radiation therapy is performed with a dose of 30 × 2 Gy(RBE) or 33 × 1.8 Gy(RBE), or for patients treated according to the hypofractionation protocol with 15 × 2.67 Gy(RBE). A possible administration of additional chemotherapy (concurrent or adjuvant) or tumor treating fields is applied in dosage and frequency according to the therapy standard outside of this study. The primary endpoint is the cumulative rate of toxicity CTC grade 2 and higher in the first 4 months. Secondary endpoints include overall survival, progression-free survival, quality of life, and neurocognition.

**Discussion:**

Aim of the GRIPS study is to prospectively assess whether the theoretical physical advantage of proton beam radiotherapy will translate into a clinical reduction of toxicity during and in the first months after therapy.

*Trial registration*

ClinicalTrials (NCT): NCT04752280.

## Background

In treatment of glioblastoma patients complete resection of the tumor should be attempted whenever possible. This should be followed by adjuvant radiotherapy up to a dose of 60 Gy and concurrent chemotherapy with temozolomide (75 mg/m^2^ KOF; daily during the radiation period) and further adjuvant chemotherapy with temozolomide (6 cycles of 150 or 200 mg/m^2^ KOF each for 5 days every 4 weeks). If surgery is not possible, the aforementioned radiochemotherapy is applied as definitive therapy.

A treatment which was investigated in the EORTC-NCIC trial but that also showed that the prognosis of glioblastoma is very unfavorable with a median survival of 14.6 months [1]. Furthermore, Glioblastomas that involve the subventricular zone appear to have a particularly unfavorable prognosis, as these have a higher risk of multifocal/distant progression [2].

Patients with a methylated MGMT promoter appear to benefit from additional administration of lomustine to radiochemotherapy with temozolomide, which significantly increased median survival in the CeTeG study up to 48.1 months [3].

While great efforts are being made to achieve treatment optimization on the drug side, radiotherapy as a standard therapy has not changed significantly in recent decades, apart from a reduction in treatment time in elderly patients with glioblastomas [4]. However, radiotherapy remains an integral part of the primary treatment of glioblastoma.

Proton beams, which have a steeper dose gradient compared to (conventional) photons, can therefore be applied in a more concentrated manner with significantly lower dose exposure outside the irradiated volume. This physical advantage could be demonstrated for higher-grade gliomas and glioblastomas in a plan comparison study [5]. Additionally, in a matched pair analysis of the Heidelberg patient collective with 66 patients with higher-grade glioma or glioblastoma who had received part of the irradiation with protons (10 Gy proton boost after 50 Gy photons) were compared with a corresponding cohort, who had received the entire irradiation with photons. While identically effective, there was a trend towards increased toxicity in the group treated only with photons over the therapy period and the first 3 months thereafter (toxicity grade 2/3: photons: 14 patients, photons + protons 6 patients; *p* < 0.1), whereas grade 3 toxicities occurred only in the group with photon therapy alone [6].

A reduction in irradiated brain volume may also affect the number of circulating lymphocytes. A recently published randomized phase II trial reduced the likelihood of high-grade radiation-induced lymphopenia in glioblastoma patients when using proton beam therapy compared to photon irradiation [7]. This might also influence survival due to higher probability of implementation of concurrent and adjuvant chemotherapy regimens.

Aim of the current study is to prospectively evaluate if the theoretical physical advantage of proton beam radiotherapy will translate into a clinical reduction of toxicity during and in the first months after therapy compared to classical photon irradiation without compromising efficacy in patients with glioblastoma.

## Methods/design

### Study design

The study is a multicenter, prospective, open-label, randomized, two-arm, phase III study, which will be conducted at 3 locations in Germany (Heidelberg, Marburg/Gießen, Stuttgart).

### Objectives

Patients in the study will be treated either by modern intensity modulated photon irradiation (IMRT) technique (standard arm) or by proton beam radiotherapy (experimental arm). The aim of the GRIPS study is to prospectively test if the theoretical physical advantage of proton beam radiotherapy will translate into a clinical reduction of toxicity during and in the first months after therapy.

### Endpoints

The primary endpoint is the cumulative rate of toxicity CTCAE ≥ grade 2 in the first 4 months. Secondary endpoints are progression-free survival (secondary endpoint of special interest), overall survival, acute and late toxicity, quality of life and neurocognition.

### Additional scientific program

The influence of the irradiation technique on the lymphocyte count will be evaluated in an additional scientific program.

### Inclusion criteria

Patients (Age ≥ 18 years, ECOG 0–2) with histologically confirmed glioblastoma (WHO grade 4) with indication for radiotherapy/chemoradiation.

### Exclusion criteria

Patients who are not capable of giving consent, previous radiation therapy to the brain or skull base, active medical implants for which no approval for ion irradiation exists at the time of treatment (e.g., pacemaker, defibrillator, etc.), contraindication to MRI imaging, concurrent participation in another clinical trial that could affect the results of this trial or the other trial.

### Randomization

**Fig. 1:**
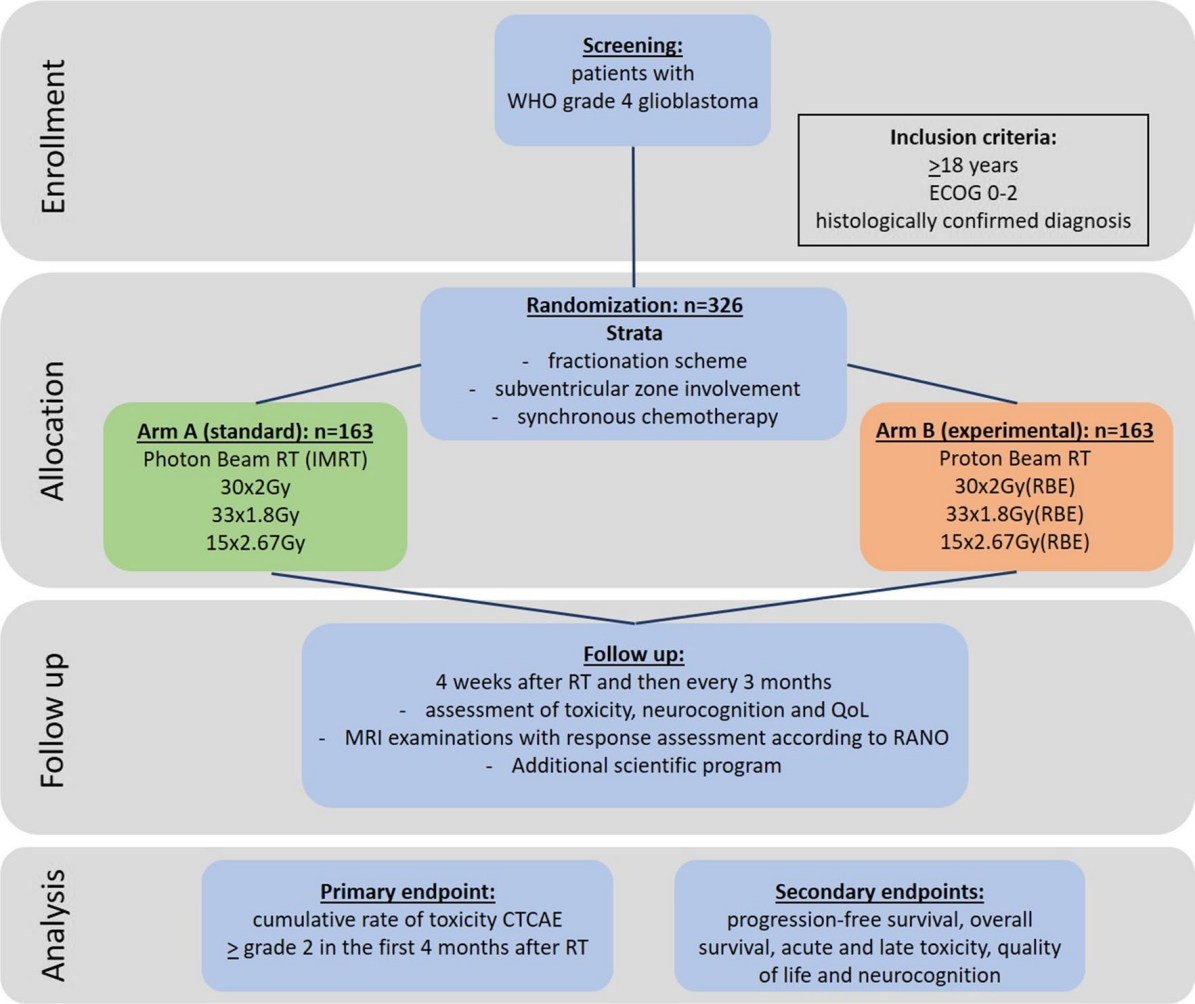
Flow diagram of the process of the study phases of the GRIPS trial. IMRT = intensity-modulated radiotherapy, RT = radiotherapy

Screened and eligible patients will be enrolled into the study after study initiation. To obtain similar distribution of prognostic variables in both treatment groups, each patient will be randomly assigned to treatment groups (1:1) in balanced permuted blocks. Stratification according to "fractionation scheme" (normofractionated/hypofractionated), "subventricular zone involvement" (yes/no) and concurrent chemotherapy (yes/no) will be applied using the web-based software randomizer.at (provided by the Institute of Medical Informatics, Statistics and Documentation of the Medical University of Graz (https://www.randomizer.at)). According to case number planning, 326 patients should be randomized, see Fig. 1.

### Target volume delineation and radiotherapy application

The patient is positioned using a suitable fixation method (e.g. thermoplastic mask). Computed tomography must be performed natively in 3 mm slice thickness or less and, if possible, also with contrast medium.

Target volume definition will be performed according to the ESTRO-ACROP guideline and additional MRI sequences [8]:

- GTV: contract-enhancing macroscopic tumor and/or resection cavity with contrast-enhancing residual tumor parts, taking into consideration planning MRI with preoperative scans and early postoperative imaging to prove for more precise delineation
- CTV: is defined as the GTV plus a 1.5–2.0 cm margin to account for microscopic spread. It should be defined considering anatomical borders and inclusion of abnormal (postoperative) T2-FLAIR signal
- PTV: CTV + 3 mm

In cases where the CTV accounts for > 25% of the total brain volume, a boost volume should be defined after 50 Gy, which covers only the GTV + 1.5–2 cm (without further consideration of abnormal T2-FLAIR signal).

Organs at risk (OAR) definitions and dose limitations for OARs are chosen according to table 2 of Niyazi et al. [8]. Expansion of OARs to create a planning risk volume (PRV) for OAR is applied when necessary and the margin should reflect the accuracy of daily set-up.

**Fig. 2:**
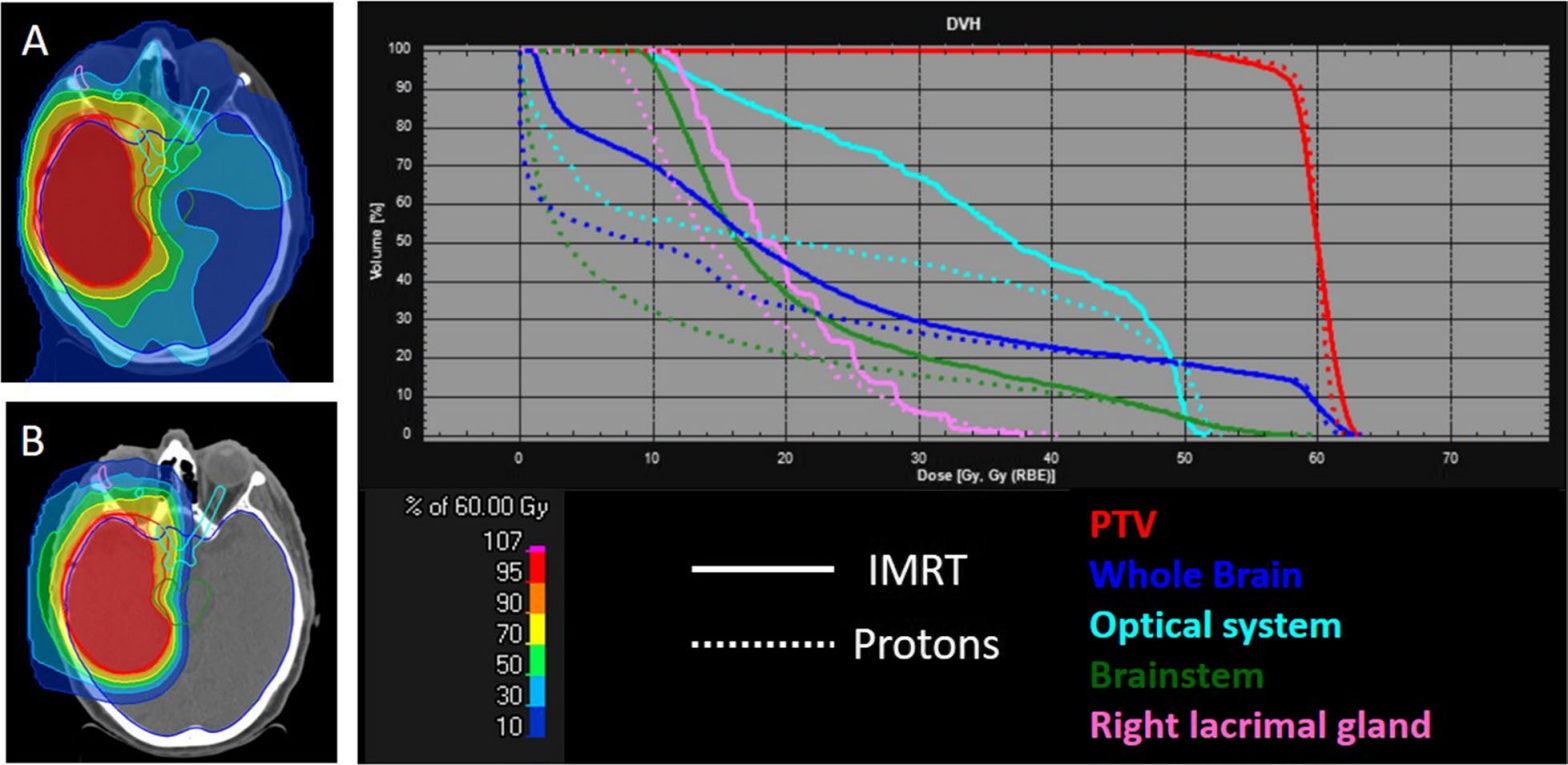
Representative transversal images show the IMRT plan (**A**) and the proton plan (**B**) of a patient with glioblastoma of the right temporal lobe. The DVH shows some of the relevant organs at risk in corresponding colors in solid and dashed lines, for IMRT and proton beam radiotherapy, respectively. While the PTV (red) coverage is similar in both techniques, dose to the surrounding organs is reduced in the proton plan

Photon radiotherapy is performed in all participating centers, while proton beam radiotherapy is performed at the Heidelberg ion beam therapy center (HIT) or Marburg ion beam therapy center (MIT) with 5 fractions per week. The applied dose is 30 × 2 Gy(RBE) or 33 × 1.8 Gy(RBE), or for patients treated according to the hypofractionation protocol ((4, 11)) due to age or comorbidities, with 15 × 2.67 Gy(RBE), all prescribed to the PTV. The dose to the PTV should be homogenous, with at least 95% of the PTV covered by the 95% prescription isodose, and dose maximum less than 107% of the prescribed dose (according to the ICRU 62 and 93 [9, 10]). Photon radiotherapy in the standard arm must be applied as IMRT. Treatment is applied with image guidance with frequency according to the location of the target volume, setup and/or the treating physician´s choice. Figure 2 shows an example of the dose distribution of a representative patient case comparing IMRT and proton radiotherapy.

A possible administration of additional chemotherapy (concurrent or adjuvant) as well as tumor treating fields is applied in dosage and frequency according to the therapy standard outside of this study. The subject of the examination within this trial is only the different radiation quality.

### Timeline of the study

The number and content of clinical visits correspond to those of standard therapy. The study-related additional effort results from the testing of neurocognition by means of the Hopkins Verbal Learning Test (HVLT-R; [11, 12]) and the regular completion of the quality-of-life questionnaires. The timeline with its different examinations is displayed in Table 1. Oncological follow-up begins 4 weeks after the end of radiotherapy. Further follow-up appointments are month 4, month 7, and subsequently every 3 months until month 25 (Table 1).

**Table 1:** Intervention and assessment schedule for the GRIPS trial

	Inclusion examination	RT	Examination after RT	V1: 4 weeks after RT	V2: month 4	V3: month 7	V4: month 10	V5: month 13	V6: month 16	V7: month 19	V8: month 22	V9: month 25
Screening	x											
CRF form	x		x	x	x	x	x	x	x	x	x	x
QLQ-C30 und QLQ-BN20	x		x		x			x				
Hopkins Verbal Learning Test- Revised	x		x		x			x				
Symptoms/ Toxicity according to CTCAE	x		x	x	x	x	x	x	x	x	x	x
MRI	x^a^			x	x	x	x	x	x	x	x	x
RT		x										
Additional scientific program	x		x	x	x							

RT = radiotherapy

Screening: includes check of inclusion and exclusion criteria, taking of medical history including surgical and pathological report, medical examination and randomization

^a^ MRI less than 4 weeks old, if older at time of inclusion, a new MRI for planning is mandatory

Patients will be recruited over a period of 4 ¼ years, and the evaluation of the study results regarding the primary endpoint of the study (toxicity grade ≥ 2) will be evaluated 4 months after the end of radiation of the last patient. The end of the study is defined as the end of the follow-up period of the last patient (25 months after start of treatment).

### Assessment of safety, toxicity and neurocognition

Acute and late radiotherapy toxicity will be assessed closely during and after therapy at each follow-up appointment using the NCI CTCAE version 5.0 criteria, quality of life will be measured using the EORTC questionnaires QLQ-C30 and QLQ-BN20. Neurocognition will be tested using Hopkins Verbal Learning Test-Revised.

### Radiological assessment

Response assessment is evaluated primarily radiologically according to the RANO criteria, see Table 1 of the publication by Wen et al. [13]. Tumor progression is defined as a new contrast uptake outside of the radiation field (beyond the high-dose region or 80% isodose line) or at least a 25% increase in tumor volume (product of two diameters perpendicular to each other). Pseudoprogression is defined as contrast uptake in the first 3 months after radiotherapy if it stabilizes on subsequent MRI scans, according to table 2 by Wen et al. [14]. Newly appeared contrast uptake originating from the ventricular zone and not located in the area of the initial contrast uptake is primarily defined as radiogenic blood brain barrier disruption. If a lesion initially defined as a pseudoprogression or as a radiogenic barrier disruption, which may turn out to be a disease progression in the further course, the date of progression is backdated. All follow up MRIs are reviewed centrally.

### Statistical considerations

The primary study objective is to demonstrate that the toxicity rate (rate of CTCAE toxicity grade ≥ 2) under proton therapy, π^Pr^, is lower than the toxicity rate under photon therapy, π^Ph^. Thus, the null hypothesis of the study is that the toxicity rate in the proton group is greater than or equal to the toxicity rate in the photon group, i.e., H_0_: ${\pi }_{A}^{Pr}\ge {\pi }_{A}^{Ph}$. For sample size planning, rates of ${\pi }_{A}^{Ph}=21\%$ for the photon group and ${\pi }_{A}^{Pr}=9\%$ for the proton group were assumed under the alternative hypothesis. This assumption results from a matched-pair analysis of the Heidelberg patient collective [6]. Under these assumptions, 276 evaluable patients (138 per group) are needed to reject the null hypothesis with a power of 1-β = 80% using the chi-square test at the one-sided significance level α = 0.025. Assuming a dropout rate of 15%, 326 patients (163 per group) will be included in the study. In the analysis, a logistic regression model will be applied for the evaluation of the primary endpoint adjusting for the confounders "fractionation," "subventricular zone involvement", and "concurrent chemotherapy". It is expected that this will increase power as compared to using the chi-square test. The secondary variables will be evaluated with methods of descriptive data analysis.

The intention-to-treat (ITT) population is the primary evaluation population for all efficacy endpoints and patient characteristics. The ITT population comprises all randomized patients which are evaluated in the treatment arm to which they were randomized. The per-protocol (PP) population includes all patients in the ITT population who received the planned therapy in its entirety and for whom documentation is complete with respect to the primary endpoint. The analyses of the PP population serve as sensitivity analyses, which will be used to investigate the robustness of the results from the ITT analysis. All patients in the ITT population in whom the planned therapy was started (at least 1 day) belong to the safety population and will be evaluated in the therapy arm to which they were treated. This is the primary evaluation population for the primary endpoint as well as for all other safety endpoints.

### Ethics

The study protocol, patient information and consent form are approved by to the local Ethics Committees (S-204/2019 for Heidelberg, B-F-2021-052 for Stuttgart and positive approval for Marburg/Gießen from 08.05.2020). The procedures set out in this trial protocol are designed to ensure that all persons involved in the trial abide by ICH harmonized tripartite guideline on Good Clinical Practice (ICH-GCP) and the ethical principles described in the applicable version of the Declaration of Helsinki. The trial will be carried out in keeping with local legal and regulatory requirements.

### Data quality assurance and data safety monitoring board

To ensure data quality and consistency, quality control measures are performed regularly. For this purpose, contents are regularly checked by a monitor in 10% of all patients included up to this point (selected at random) as part of quality assurance. Furthermore, an independent Data Safety Monitoring Board (DSMB) will monitor enrollment, reported adverse events, PFS as the primary secondary endpoint, and data quality at least annually. Based on its report, the DSMB will make recommendations to the principal investigator regarding study modification, continuation or termination. The mission of the DSMB will be to ensure the ethical conduct of the study, as well as to protect the safety interests of the patients in the study.

## Discussion

Previous published in-house data has shown that proton beam therapy as a boost concept might offer a dosimetric and clinical benefit in patients with high grade glioma and glioblastoma. This multicenter, prospective, open-label, randomized, two-arm phase III study is the first to investigate whether patients with glioblastoma experience significantly less toxicity when the whole radiotherapy is delivered with proton irradiation compared to classical photon irradiation, and whether this improves patient quality of life without compromising efficacy.

### Trial status

Protocol version 1.2d (12.08.2021). The trial started in April 2021 and is currently recruiting. Length of clinical phase approximately 66 months with planned end of the study in Q3 2025 (last patient in) and Q4 2027(end of follow up).

## Data Availability

The data used in this analysis is from publications available in the public domain.
